# Sensing, Uptake and Catabolism of L-Phenylalanine During 2-Phenylethanol Biosynthesis *via* the Ehrlich Pathway in *Saccharomyces cerevisiae*

**DOI:** 10.3389/fmicb.2021.601963

**Published:** 2021-02-25

**Authors:** Jun Dai, Huili Xia, Chunlei Yang, Xiong Chen

**Affiliations:** ^1^Key Laboratory of Fermentation Engineering (Ministry of Education), National “111” Center for Cellular Regulation and Molecular Pharmaceutics, Hubei Provincial Cooperative Innovation Center of Industrial Fermentation, College of Bioengineering, Hubei University of Technology, Wuhan, China; ^2^ABI Group, College of Marine Science and Technology, Zhejiang Ocean University, Zhoushan, China; ^3^State Key Laboratory of Biocatalysis and Enzyme Engineering, School of Life Sciences, Hubei University, Wuhan, China; ^4^Tobacco Research Institute of Hubei Province, Wuhan, China

**Keywords:** 2-phenylethanol, sensing of L-phenylalanine, uptake of L-phenylalanine, Ehrlich pathway, *Saccharomyces cerevisiae*

## Abstract

2-Phenylethanol (2-PE) is an important flavouring ingredient with a persistent rose-like odour, and it has been widely utilized in food, perfume, beverages, and medicine. Due to the potential existence of toxic byproducts in 2-PE resulting from chemical synthesis, the demand for “natural” 2-PE through biotransformation is increasing. L-Phenylalanine (L-Phe) is used as the precursor for the biosynthesis of 2-PE through the Ehrlich pathway by *Saccharomyces cerevisiae*. The regulation of L-Phe metabolism in *S. cerevisiae* is complicated and elaborate. We reviewed current progress on the signal transduction pathways of L-Phe sensing, uptake of extracellular L-Phe and 2-PE synthesis from L-Phe through the Ehrlich pathway. Moreover, the anticipated bottlenecks and future research directions for *S. cerevisiae* biosynthesis of 2-PE are discussed.

## Introduction

2-Phenylethanol (2-PE) is a higher alcohol with a rose-like odour. 2-PE contributes significantly to the flavour and aroma of beer, bread, cocoa, cheese, soy sauce, and other fermented foods and has been widely used in the perfume, cosmetics, and food industries (Chung et al., 2000; Stark et al., 2002). 2-PE is also the precursor for the production of 2-phenylethyl acetate (2-PEA), which is an important flavouring agent with floral and rose-like odours (Carlquist et al., 2015). Moreover, 2-PE is utilized in sanitation and hygiene products, which mainly rely on its antifungal and antibacterial characteristics (Etschmann et al., 2002). Currently, the Flavour and Extract Manufacturers Association (FEMA), the Food and Drug Administration (FDA), the Joint Expert Committee on Food Additives (JECFA), the Council of Europe (COE), and other international organizations have approved the use of 2-PE as a flavouring agent in food (Scognamiglio et al., 2012).

At present, the global market demand for 2-PE is increasing every year, with an annual global demand of 1,000 tons in 2011, equal to a market value of $700 million (Hua and Xu, 2011). Currently, most 2-PE is synthesized by chemical methods, including the Friedel-Craft reaction of ethylene oxide with benzene, catalytic reduction of styrene oxide, and oxidation of propylene with 2-phenylethyl hydroperoxide (Martínez-Avila et al., 2018). This process involves high temperatures (>300°C) and toxic chemicals (benzene and styrene) and leads to the restricted availability of 2-PE (Chreptowicz et al., 2016; Martínez-Avila et al., 2018). Concerning environmental issues and health hazards, 2-PE from chemical synthesis is less preferred or restricted in the food and cosmetics industries. Although 2-PE is found naturally in some plants, such as rose, hyacinths, and jasmine, and natural 2-PE can be extracted from essential oils of flowers, the volume of natural 2-PE extracted from flowers is limited, and the market price of 2-PE is as high as $1,000/kg (Hua and Xu, 2011).

The United States Food and Drug Administration and European legislation have determined that 2-PE from microbial synthesis is considered “natural” (Hua and Xu, 2011). Therefore, the biotransformation of natural 2-PE has received increasing attention and may be the most effective alternative (Lukito et al., 2019). Previous studies have demonstrated that *Saccharomyces cerevisiae*, *Kluyveromyces marxianus*, *Kluyveromyces lactis*, *Pichia fermentans*, *Pichia anomala*, *Schizosaccharomyces pombe*, *Yarrowia lipolytica*, *Zygosaccharomyces rouxii*, and *Hansenula anomala* can synthesize 2-PE from L-phenylalanine (L-Phe) or glucose through the Ehrlich pathway and phenylpyruvate (PPA) pathway (Romagnoli et al., 2015; Martínez-Avila et al., 2018; Cordente et al., 2019; Hassing et al., 2019).

Although microorganisms possess the ability to synthesize 2-PE from glucose through the PPA pathway, the process is very complex, with many metabolic branches competing for carbon flow. In addition, 2-PE itself is toxic to microbial cells, and 2-PE biosynthesis is strongly feedback-inhibited by L-Phe. Therefore, the efficiency of the phenylpyruvate pathway is very low (Hassing et al., 2019).

2-Phenylethanol can also be efficiently synthesized from L-Phe through the Ehrlich pathway, which consists of three steps, transamination, decarboxylation, and reduction (Hazelwood et al., 2008). To improve 2-PE production, various methods have been employed, including strain mutagenesis and selection, medium composition and culture condition optimization, and *in situ* product removal techniques (Hua and Xu, 2011; Martínez-Avila et al., 2018; Qian et al., 2019; Wang et al., 2019). In this review, we focus on the regulation of the sensing, transportation, and catabolism of L-Phe to produce 2-PE.

## Signal Transduction of L-Phe

Yeast cells can use various amino acids for growth. To discriminate amino acids, *S. cerevisiae* has evolved to have a complete extracellular amino acid-sensing system [Ssy1-Ptr3-Ssy5 signalling sensor system or Ssy1-Ptr3-Ssy5 (SPS) sensor system] and intracellular amino acid-sensing system [target of rapamycin (TOR) pathway], which are crucial for sensing extracellular and intracellular amino acids, respectively (Klasson et al., 1999; Forsberg and Ljungdahl, 2001; Schneper et al., 2004; Zhang et al., 2018).

### External L-Phe Sensing by the SPS Sensor System

**Figure 1:**
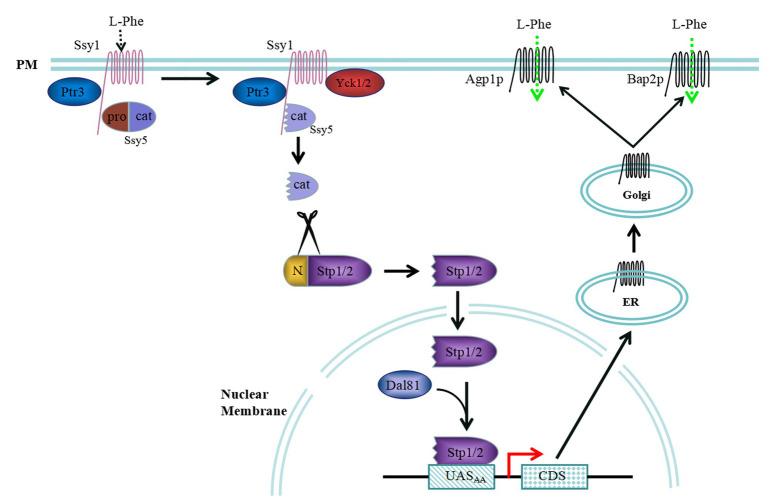
Extracellular L-Phenylalanine (L-Phe) signalling pathway mediated by the Ssy1-Ptr3-Ssy5 (SPS) sensor system. When L-Phe is the sole nitrogen source, Ssy1 may react to it and recruit casein kinase Yck1/2 to phosphorylate the N-terminus of Ssy5. The N-terminus of Ssy5 is ubiquitinated and degraded to free the Cat domain, which cleaves the N-terminal regulatory domains of Stp1 and Stp2. The shorter forms of Stp1 and Stp2 target the nucleus and bind the UAS_AA_ elements *AGP1* and *BAP2*, activating expression of these genes. Then, Agp1p and Bap2p are secreted into the membrane of the endoplasmic reticulum, where they are processed, modified, and transferred to the Golgi apparatus for further processing and packaging. Finally, Agp1p and Bap2p are localized to the cell membrane and transport extracellular L-Phe into cells.

Extracellular amino acids can be detected *via* the SPS sensor pathway in *S. cerevisiae*, and some amino acid permeases are regulated by the SPS sensor pathway. The sensing of extracellular amino acids is mostly controlled by the signal transduction pathway, which in turn, regulates the dynamic interactions between transcription factors and specific promoter binding sites (Figure 1). The SPS sensor system is a plasma membrane (PM)-localized complex consisting of three core components, Ssy1, Ptr3, and Ssy5 (Forsberg and Ljungdahl, 2001). Ssy1 is an important component of the PM that exhibits high sequence similarity with amino acid permease families (Didion et al., 1998; Gaber et al., 2003). However, Ssy1 differs from amino acid permeases because of its long cytoplasmically oriented N-terminal domain and inability to transport amino acids. In addition, Ssy1 serves as an extracellular amino acid receptor and a scaffold that concatenates Ptr3 and Ssy5, as well as other membrane proteins, through its long cytoplasmically oriented N-terminal domain (Iraqui et al., 1999b; Klasson et al., 1999).

Ssy5, a core component of the SPS, is a serine protease expressed as an inactive zymogen that contains a regulatory N-terminal pro-domain and noncovalently bound C-terminal catalytic-domain (Martins et al., 2018). The endoprotease activity of Ssy5 is inhibited by its own regulatory N-terminal pro-domain. In response to extracellular amino acids, Ssy5 undergoes an autocatalytic event upon proteasomal degradation of its regulatory N-terminal pro-domain, leading to the activation of its endo-protease activity (Martins et al., 2019). Once an extracellular amino acid signal is received, the Ssy1 conformation is altered, which recruits the casein kinase Yck1/2. The N-terminal pro-domain of Ssy5 is phosphorylated by Yck1/2, as facilitated by Ptr3, which is then modified by the ubiquitin ligase complex. Finally, the pro-domain is degraded by the 26S proteasome, freeing the Cat domain (Abdel-Sater et al., 2011; Omnus and Ljungdahl, 2013).

The transcription factors Stp1 and Stp2 are cleaved by the freed Cat domain, and the shortened Stp1 and Stp2 peptides are translocated into the nucleus, where they bind to the promoter region of the SPS regulatory gene to induce transcription (Tumusiime et al., 2011; Omnus and Ljungdahl, 2014). Stp1 and Stp2 are initially produced with N-terminal regulatory domains, preventing them from entering the nucleus. Stp1 and Stp2 are homologous 10-kDa zinc finger transcription factors that serve as downstream effectors of the SPS sensor system, and their N-terminal domains are crucial for their activity (Andréasson and Ljungdahl, 2002). Stp1 and Stp2 possess two regulatory motifs, I (RI) and II (RII), and RII has an endoprotease-processing site that is required for the cleavage of Stp1 between cysteine 85 and serine 86 by the Ssy5 Cat-domain (Andréasson and Ljungdahl, 2004; Omnus et al., 2016). Upon amino acid induction, the Stp1/2 N-terminal regulatory domains are degraded by the SPS sensor controller Ssy5 signalling protease, and the shorter Stp1/2 are then localized to the nucleus, where they bind to the specific upstream activation element UAS_AA_ of the targeted genes, namely, *AGP1*, *BAP2*, *BAP3*, *GNP1*, *DIP5*, and *MUP1*. However, complete Stp1 and Stp2 are widely distributed in the cytoplasm and cannot enter the nucleus in the absence of amino acids.

When L-Phe is used as the sole nitrogen source to produce 2-PE, Ssy1 located in the plasma membrane may react with it and recruit casein kinase Yck1/2 to phosphorylate the Ssy5 N-terminus. The Ssy5 N-terminus is then ubiquitinated and degraded to free the Cat domain, which cleaves the N-terminal regulatory domains of Stp1 and Stp2. The shorter forms of Stp1 and Stp2 pass through the nuclear membrane and bind to the UAS_AA_ elements *AGP1*, *BAP2*, and *BAP3*, inducing the expression of these three genes. Agp1p, Bap2p, and Bap3p are secreted into the endoplasmic reticulum membrane adjacent to the nuclear membrane, where they are processed and modified and then transferred to the Golgi apparatus for further processing and packaging. Finally, Agp1p, Bap2p, and Bap3p are localized to the cell membrane and transport extracellular L-Phe into cells (Figure 1).

In addition, a small amount of complete Stp1 and Stp2 can leak into the nucleus, where they can bind to the upstream activation sequence of a target gene to facilitate Dal81/Uga35 function. However, the nuclear membrane protein Asi1-3 localized to the nuclear membrane plays important roles in the cytoplasmic retention of Stp1 and Stp2 and can prevent the complete Stp1 and Stp2 proteins from entering the nucleus (Zargari et al., 2007).

### Intracellular Amino Acid Sensing by the TOR Pathway

**Figure 2:**
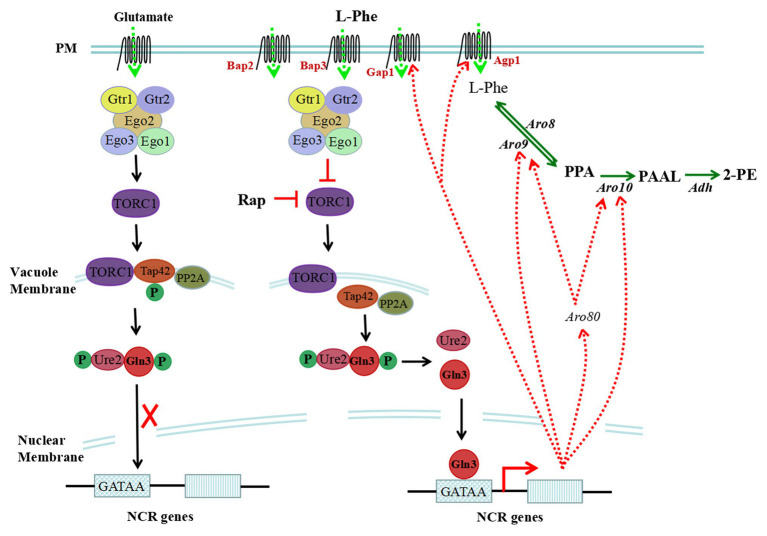
Sensing and regulation of intracellular L-Phe levels through the target of rapamycin (TOR) pathway. The TOR signalling cascade includes the EGOC complex, TOR complex 1 (TORC1), and downstream effectors (Tap42-PPase and Sch9). Intracellular L-Phe is sensed by the EGOC complex, and TORC1 can be inhibited. However, upon rapamycin treatment or in the presence of a poor nitrogen source, the activity of TORC1 can be restrained, which results in the dephosphorylation of Tap42, freeing it from the vacuole membrane. Gln3 is dephosphorylated by freed Tap42 and is freed from Ure2; then, it targets the nucleus and binds to the GATAA/G motif of nitrogen catabolite repression (NCR)-sensitive genes, activating the transcription of NCR-sensitive genes. In the presence of glutamate, TORC1 is activated and Gln3 cannot be dephosphorylated and resides in the cytoplasm, which represses the expression of NCR-sensitive genes.

In eukaryotic cells, the TOR signalling pathway controls cell growth and proliferation. Yeast cells recognize intracellular amino acid conditions through the TOR-sensing pathway, which responds to the availability of amino acids (Schneper et al., 2004). The TOR signalling cascade includes the EGOC complex, an upstream regulatory element, TOR complex 1 (TORC1), and downstream effectors (Tap42-PPase and Sch9). TORC1 is inhibited by rapamycin and is structurally and functionally conserved. As the core component of the TOR signalling pathway, TORC1 is composed of the Tor1, Kog1, Tco89, and Lst8 proteins (Reinke et al., 2004), and TORC1 primarily recognizes the amino acid/nitrogen conditions in yeast cells. Upon nitrogen starvation/reduction or rapamycin treatment, TORC1 activity is restrained. An increase in nitrogen or cycloheximide treatment results in the activation of TORC1 (Binda et al., 2009). The intracellular amino acid signal is transmitted to the related protein of TORC1 through the upstream component EGOC to activate or inhibit the activity of TORC1 (Figure 2).

EGOC includes structural subunits and regulatory subunits in *S. cerevisiae*, and the structural subunits are EGO/Rag complexes consisting of Ego1, Ego2, and Ego3 (Dubouloz et al., 2005; Powis et al., 2015). EGOC is bound to a vacuole membrane by Ego1. The regulatory subunit consists of Gtr1 and Gtr2, and the nucleotide-binding status of Gtr1 and Gtr2 regulates the activity of TORC1 (Binda et al., 2009; Zhang et al., 2018). Upon binding to GTP, Gtr1 interacts with the Tco89 and Kog1 proteins, and TORC1 is activated to block the utilization of poor nitrogen sources, which is hindered by leucine starvation (Kim et al., 2008; Broach, 2012; Powis et al., 2015). The nucleotide binding state of Gtr1 is regulated by several regulatory factors, including guanine nucleotide exchange factor (GEF; Sancak et al., 2008; Binda et al., 2009), GTPase activating protein (GAP; Powis and De Virgilio, 2016), and the Seh1-associated complex proteins Npr2 and Npr3 (Neklesa and Davis, 2009). These regulatory factors are sensitive to the levels of intracellular nitrogen and amino acids and activate or inhibit the activity of TORC1 to modulate intracellular nitrogen and amino acid metabolism through the downstream regulatory factors Tap42 and Sch9. Intracellular glutamate, sensed by the EGOC complex, activates TORC1 and represses the expression of nitrogen catabolite repression (NCR)-sensitive genes in *S. cerevisiae*. However, L-Phe is a nonpreferred nitrogen source for *S. cerevisiae* and is sensed by EGOC, which may restrain the activity of TORC1 and derepress the expression of NCR-sensitive genes (Figure 2).

### Regulation of NCR *via* the TOR Signalling Pathway

*Saccharomyces cerevisiae* exhibits a hierarchical preference for nitrogen sources, which are usually classified as preferred nitrogen sources and poor nitrogen sources. *Saccharomyces cerevisiae* cultured in the presence of nitrogen sources with different qualities presents sequential utilization of preferred, intermediate, and poor nitrogen sources, which is controlled by NCR (Henschke and Jiranek, 1993; Boer et al., 2007). The preferred nitrogen sources include ammonium salts, glutamate, glutamine, asparagine, and other nitrogen sources. The poor nitrogen sources include methionine, proline, allantoin, γ-aminobutyric acid, urea, and other nitrogen sources (Magasanik and Kaiser, 2002; Godard et al., 2007). However, leucine and phenylalanine are considered to be “intermediate” nitrogen sources (Boer et al., 2007). The rough classification of nitrogen sources is generally based on the following two criteria: the extent to which an individual nitrogen source supports growth when it is the sole nitrogen source and the extent to which a nitrogen source prevents the utilization of poorer nitrogen sources (Magasanik and Kaiser, 2002).

The classification of nitrogen sources and the priority of nitrogen source assimilation vary. Currently, the order of L-Phe assimilation has not been clearly established and differs between different yeast strains and environmental conditions. In a previous study, when different brewing and wine yeast strains were cultured in anaerobic synthetic medium that mimicked grape must supplied with various nitrogen compounds, L-Phe was consumed early along with aspartate, threoine, glutamate, histidine, methionine, serine, and glutamine. Ammonium and tryptophan were consumed late (Crépin et al., 2012).

However, based on transcriptomic analysis, L-Phe is considered a nonpreferred nitrogen source. When *S. cerevisiae* ∑1278b was grown in aerobic minimal buffer medium with glucose and 21 different nitrogen sources as the sole nitrogen source, L-Phe supported slower growth and exerted a weaker NCR effect. *Saccharomyces cerevisiae* CEN. PK113-7D was grown in aerobic glucose-limited chemostat cultures with various amino acids as nitrogen sources, and L-Phe exerted an “intermediate” NCR response. There was no direct correlation between the growth rate of each nitrogen source and the degree of NCR (Boer et al., 2007; Godard et al., 2007).

NCR is modulated by four GATA family transcription factors, including the transcriptional activators Gln3/Gat1 and transcriptional repressors Dal80/Gzf3. In the presence of preferred nitrogen sources, Gln3 and Ure2 form complexes in the cytoplasm, which repress the transcription of NCR-sensitive genes. However, in the presence of nonpreferred nitrogen sources, limited nitrogen or added rapamycin, Gln3 is dephosphorylated and freed. Then, it is targeted to the nucleus and binds to the GATAA/G motifs of NCR-sensitive gene promoters, activating gene transcription (Beck and Hall, 1999; Cox et al., 2000; Kulkarni et al., 2001; Tate et al., 2018).

Tap42, an important downstream effector of TORC1, is necessary for the dephosphorylation of Gln3. The extent of Gln3 phosphorylation is synergistically affected by Tap42 and protein phosphatase 2A (PP2A; Beck and Hall, 1999). In the presence of rich nitrogen sources, the activation of TORC1 results in the phosphorylation of Tap42, which combines with PP2A to form a complex located in the vacuole membrane. When Gln3 cannot be dephosphorylated and resides in the cytoplasm, NCR-sensitive genes cannot be expressed (Shamji et al., 2000; Duvel et al., 2003). In the presence of nonpreferred nitrogen sources, limited nitrogen or added rapamycin, TORC1 is inhibited, resulting in the dephosphorylation of Tap42, freeing it from the vacuole membrane. When Gln3 is dephosphorylated by freed Tap42, it is localized to the nucleus and binds to the GATAA/G motifs of NCR-sensitive genes, activating their transcription. In addition to the Tap42-PP2A complex, Ure2 also affects the subcellular localization of Gln3. Ure2 acts as the anchor of Gln3 to maintain its residence in the cytoplasm (Broach, 2012). When Ure2 is inactivated, Gln3 constitutively targets the nucleus (Salmon and Barre, 1998).

The genes related to L-Phe metabolism and regulated by NCR include the permeases *GAP1* and *AGP1*, aromatic aminotransferase *ARO9*, phenylpyruvate decarboxylase *ARO10*, aromatic amino acid transcription factor *ARO80*, and NAD-dependent glutamate dehydrogenase *GDH2*. With L-Phe as the sole nitrogen source or added rapamycin, the expression levels of these genes can be upregulated.

## Uptake of Extracellular L-Phe

Amino acids are important nitrogen-containing compounds and play central roles in growth and metabolism. Amino acid transporter (AAP) families with conserved sequences and architectural characteristics are critical for the transportation of amino acids (Cain and Kaiser, 2011; Wong et al., 2012). Amino acid permeases driven by proton gradients constitute the largest nitrogen source transport system in *S. cerevisiae* and play central roles in nitrogen metabolism and protein synthesis (Horák, 1997; Zhang et al., 2019). To date, 24 AAPs have been reported in *S. cerevisiae*, and each of them consists of approximately 600 amino acids (Grauslund et al., 1995; Zhu et al., 1996). In addition, these transporters share a similar conformation comprising 12 transmembrane domains and cytoplasmically oriented N-terminal and C-terminal domains (Grauslund et al., 1995). These transporters are critical for transporting amino acids and other amines. Permeases that have been reported to transport L-Phe through the PM include Agp1p, Bap2p, Bap3p, and Gap1p (Table 1; Sáenz et al., 2014; Zhang et al., 2019).

**Table 1:** Plasma membrane (PM)-localized transporters of L-Phe.

Permease	Substrate(s)	Binding motifs	Regulation pattern(s)	References
Agp1p	Broad substrate range (general amino acid)	GATA, UAS_AA_	NCR, SPS	Schreve et al., 1998; Iraqui et al., 1999b; Abdel-Sater et al., 2004
Gap1p	Broad substrate range (L-amino acids, D-amino acids)	GATA	NCR	Jauniaux and Grenson, 1990; André et al., 1993; Van Zeebroeck et al., 2009
Bap2p	branched-chain amino acids (Leu, Ile, Val)	Leu3p binding site, GAGTCA, UAS_AA_	SPS, GAAC	Grauslund et al., 1995; Didion et al., 1996; Nielsen et al., 2001
Bap3p	branched-chain amino acids (Leu, Ile, Val)	UAS_AA_	SPS	Didion et al., 1998

Agp1p encoded by the *AGP1* gene is a general amino acid permease and has a broad substrate range and low substrate affinity. Earlier research found that asparagine and glutamine are the major substrates of Agp1p in *S. cerevisiae* YCC5, with K_m_ < 1.0 mM. Moreover, Agp1p can transport L-Phe and other uncharged amino acids when these amino acids are present in millimolar concentrations (Schreve et al., 1998). Iraqui found that L-Phe can be effectively transported by Agp1p and that *SSY1* is required for the transcriptional induction of the *AGP1* gene (Iraqui et al., 1999b). Agp1p may be regulated according to the L-Phe titre and/or that of other nitrogen sources, and Agp1p is regulated simultaneously by the SPS-sensing pathway and NCR. The *AGP1* promoter region has a *cis*-sequence called UAS_AA_ and several 5'-GATA-3' motifs, which can bind to Stp1 and GATA family transcription factors separately, activating transcription of the *AGP1* gene (Schreve et al., 1998; Iraqui et al., 1999b; Abdel-Sater et al., 2004; Tate et al., 2010). The UAS_AA_ element consists of two inversely repetitive 5'-CGGC-3' motifs separated by six nucleotides.

When *S. cerevisiae* is grown with L-Phe as the sole nitrogen source to produce 2-PE, the cells recognize extracellular L-Phe through the SPS-sensing pathway. The signal is transmitted to the downstream effector factor Stp1, which then passes through the nuclear membrane and binds the UAS_AA_ elements of *AGP1*, inducing the expression of *AGP1* genes (Figure 1). Extracellular L-Phe can be transported into the cytoplasm and serves as a non-preferred nitrogen source to exert a weak NCR effect sensed by the TOR pathway. In addition, upon rapamycin treatment or in the presence of a poor nitrogen source, the activity of TORC1 is restrained. Gln3 is dephosphorylated by the downstream effector factor of TOR and targets the nucleus, where it binds to 5'-GATA-3' motifs, activating transcription of the *AGP1* gene (Figure 2).

Gap1p is also a broad-range nitrogen source transporter that can transport all natural L-amino acids, such as L-Phe, some D-amino acids, γ-aminobutyric acid, and polyamines. Transcription of the *GAP1* gene in *S. cerevisiae* is mainly regulated by the NCR pathway and the general amino acid control pathway (GAAC pathway) and can be inhibited in the presence of ammonium salts (Jauniaux and Grenson, 1990; André et al., 1993; Van Zeebroeck et al., 2009).

Bap2p is a branched-chain amino acid permease that mainly transports leucine, isoleucine, and valine with high efficiency and high affinity (Grauslund et al., 1995). Bap2p can also transport L-Phe. Moreover, when *S. cerevisiae* Y294 was grown with ammonium as the sole nitrogen source and the cells were harvested and transferred to a buffer system containing L-Phe and leucine, Bap2p was the major L-Phe transporter in this specific environment, where extracellular leucine is an important trigger for the induction of *BAP2* gene transcription (Didion et al., 1996; Sáenz et al., 2014). The *BAP2* promoter contains a Leu3p-binding site, one or two Gcn4p-binding sites (GAGTCA motif) and a UAS_AA_ motif, and transcription of the *BAP2* gene is regulated by Leu, the SPS sensor system and the GAAC pathway (Grauslund et al., 1995; Nielsen et al., 2001). In addition to the three amino acids mentioned above, Bap3p can also transport L-Phe. Bap3p is a branched-chain amino acid permease that is very similar to Bap2p and has high affinity for branched-chain amino acids. However, the regulation of *BAP3* gene transcription is controlled by the SPS sensor system (Didion et al., 1998).

## 2-PE Synthesis *Via* The Ehrlich Pathway

### Major Enzymes of the Ehrlich Pathway

**Figure 3:**
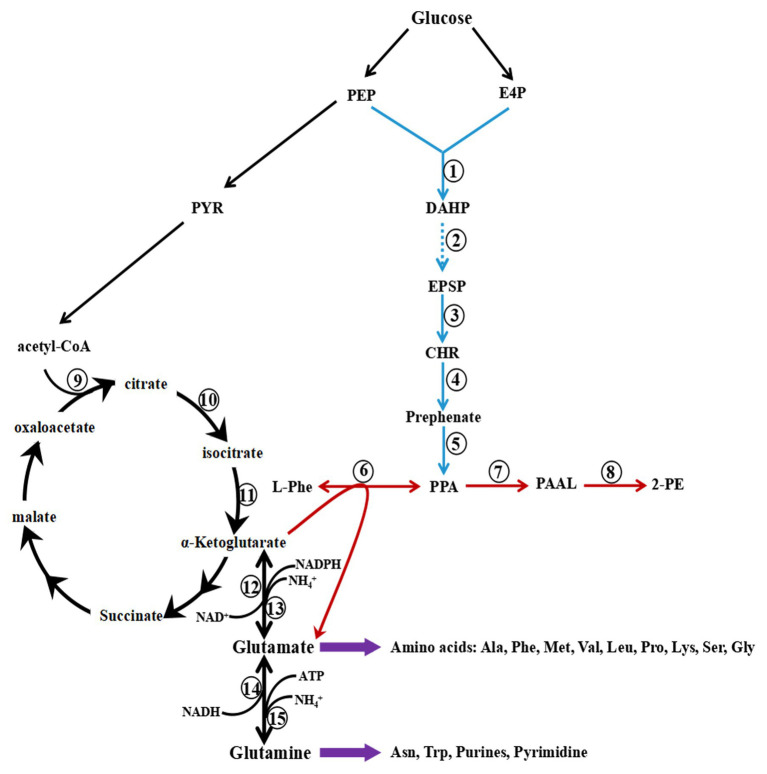
Metabolic pathway of 2-Phenylethanol (2-PE) production in *Saccharomyces cerevisiae*. The Ehrlich pathway (red) and phenylpyruvate pathway (the combination of red and blue) produce 2-PE. PEP, phosphoenolpyruvate; PYR, pyruvate; E4P, erythrose-4-phosphate; DAHP, 3-deoxy-D-arabinoheptulosonate; DHQ, 3-dehydroquinate; DHS, 3-dehydroshikimate; SHK, shikimate; S3P, shikimate-3-phosphate; EPSP, 5-enolpyruvyshikimate-3-phosphate; CHR, chorismite; L-Phe, L-phenylalanine; PPA, phenylpyruvate; PAAL, phenylacetaldehyde; and 2-PE, 2-phenylethanol. 1, phospho-2-dehydro-3-deoxyheptonate aldolase *ARO3/4*; 2, pentafunctional AROM polypeptide *ARO1*; 3, chorismate synthase *ARO2*; 4, chorismate mutase *ARO7*; 5, prephenate dehydratase *PHA2*; 6, aminotransferase *ARO8/9*; 7, decarboxylase *ARO10*; 8, dehydrogenase *ADH*; 9, citrate T-cell target antigen *CTT1/2*; 10, aconitase *ACO1*; 11, isocitrate dehydrogenase *IDH1/2*; 12, NAD-dependent glutamate dehydrogenase 2 *GDH2*; 13, NADP-dependent glutamate dehydrogenase 3 *GDH3*; 14, NADH-dependent glutamate synthase 1 *GLT1*; and 15, glutamate-ammonia ligase *GLN1*.

Two pathways in *S. cerevisiae* lead to the synthesis of 2-PE, the phenylpyruvate pathway and the Ehrlich pathway (Figure 3). When L-Phe is the precursor, 2-PE is synthesized by the Ehrlich pathway, which consists of three steps: conversion of L-Phe to phenylpyruvate by aromatic aminotransferases, decarboxylation of phenylpyruvate to phenylacetaldehyde (PAAL) by phenylpyruvate decarboxylase, and finally, reduction of PAAL to 2-PE by alcohol dehydrogenase (Hazelwood et al., 2008; Qian et al., 2019). Two isoenzymes are involved in the first step, aminotransferases I and II, which are encoded by *ARO8* and *ARO9*, respectively. *ARO8* is constitutively expressed and regulated by the general control of the amino acid biosynthesis pathway (Iraqui et al., 1998).

The expression level of *ARO9* is upregulated in the presence of aromatic amino acids (L-Phe, tryptophan, or tyrosine), poor nitrogen sources (urea or proline) or the addition of rapamycin. When *S. cerevisiae* is cultured in the presence of preferred nitrogen sources, the transcription of *ARO9* is low or negligible (Iraqui et al., 1999a; Godard et al., 2007). The enzymes that catalyse the second step are thiamine diphosphate-dependent decarboxylases, including Aro10p, Pdc5p, Pdc6p, Pdc1p, and Thi3p. When L-Phe is the sole nitrogen source, Aro10p is the primary decarboxylase catalyst for the decarboxylation of phenylpyruvate to produce phenylacetaldehyde (Vuralhan et al., 2005). Similar to the expression of *ARO9*, the transcription of *ARO10* is low or negligible in the presence of preferred nitrogen sources. *ARO10* is induced by aromatic amino acids (L-Phe, tryptophan, or tyrosine), poor nitrogen sources (urea or proline) or the addition of rapamycin (Eden et al., 2007).

In the Ehrlich pathway, the flux from L-Phe to phenylacetaldehyde limits the efficiency of 2-PE biosynthesis. Therefore, the production of 2-PE can be effectively elevated by overexpression of the crucial genes of the Ehrlich pathway, *ARO8*, *ARO9*, and *ARO10*. It has been demonstrated that overexpression of the *ARO9*, *ARO10*, and *ARO80* genes in *S. cerevisiae* W303-1B results in an increased 2-PE titre. Coexpression of the *ARO9*, *ARO10*, and *ARO80* genes and disruption of *ALD3* results in a yield of 2-PE of 4.8 g/L (Kim et al., 2014). Subsequently, in *S. cerevisiae* S288c, overexpression of *ARO8* and *ARO10* increases the yield of 2-PE by 9.3 and 16.3%, respectively, and coexpression of *ARO8* and *ARO10* leads to an increase in the yield of 2-PE by 36.8% (Yin et al., 2015).

The last step of the Ehrlich pathway involves the reduction of phenylacetaldehyde to 2-PE by alcohol dehydrogenases. The main genes encoding alcohol dehydrogenases are *ADH1*, *ADH2*, *ADH3*, *ADH4*, and *ADH5*. The final reaction can be catalysed by any of these alcohol dehydrogenases (Dickinson et al., 2003). However, although the overexpression of different alcohol dehydrogenase genes did not affect the efficiency of 2-PE synthesis in *S. cerevisiae* YPH499, coexpression of *ADH* and *ARO10* increased the concentration of 2-PE by 6.5-fold (Shen et al., 2016). In addition, phenylacetaldehyde is competitively oxidized to phenylacetic acid. The ratio of acid to alcohol depends on the cellular redox status and cultivation conditions. Under anaerobic conditions, *S. cerevisiae* generates excess NADH, resulting in the final reduction step of the Ehrlich pathway, favouring the synthesis of 2-PE (Vuralhan et al., 2003). 2-PE synthesis efficiency can also be improved by eliminating the competitive pathway or increasing the supply of the cofactor NADH (Kim et al., 2014). In addition, deficiency of NADH and/or L-Phe may limit the efficiency of 2-PE synthesis in the Ehrlich pathway. It has been demonstrated that when *GDH2*, *GAP1*, *ARO8*, *ARO10*, and *ADH2* were coexpressed in *S. cerevisiae* YS58, the intracellular level of L-Phe was increased and NADH was regenerated, leading to the concentration of 2-PE increasing to 6.3 g/L (Wang et al., 2018).

### Cis/Trans-Acting Regulatory Factors of the Ehrlich Pathway

**Figure 4:**
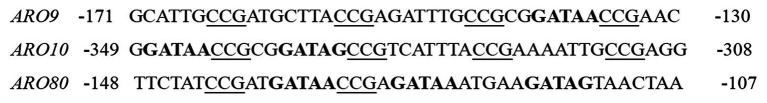
Promoter sequences of Aro80 target genes. CCG triplets, the binding sites of Aro80, are underlined, and potential GATA factor binding sites (GATAA/G) are indicated in bold.

In the Ehrlich pathway, *ARO9* and *ARO10* encode the crucial enzymes of L-Phe metabolism, and their expression levels affect 2-PE production. The expression of *ARO9/10* is synergistically regulated by GATA factors and Aro80, but the details of the regulatory mechanism are unclear. The promoters of *ARO9*, *ARO10*, and *ARO80* contain GATA motifs, which are the binding sites for Gln3 and Gat1 (Figure 4; Eden et al., 2007). In the presence of a poor nitrogen source or upon rapamycin treatment, Gln3 is freed from the Ure2 protein and targets the nucleus, binding to the GATAA/G motifs of the *ARO9*, *ARO10*, and *ARO80* promoter genes to activate transcription. In addition to the binding site for Gln3, the *ARO9*, *ARO10*, and *ARO80* gene promoters have Aro80 binding sites consisting of four CCG repeats separated by 7 bp. Aro80 constitutively binds to a pair of adjacent CCG motifs with different orientations and spacings, and the binding state is not affected by intracellular aromatic amino acids (Macpherson et al., 2006). Aro80, a member of the Zn_2_Cys_6_ family of proteins, can activate the expression of *ARO9* and *ARO10* in the presence of aromatic amino acids. The expression of *ARO9* and *ARO10* is synergistically regulated by Aro80 and Gln3/Gat1, respectively. Gln3/Gat1 indirectly affects the activity of Aro80, which is required for the binding of Gln3/Cat1 to the *ARO9* and *ARO10* promoter genes (Lee and Hahn, 2013).

Compared with modifications of the core genes of the Ehrlich pathway, modifications of regulatory factors are simple and efficient alternatives that can activate or inhibit the expression of multiple genes simultaneously. Aro80 and GATA can regulate transcription of the crucial genes *ARO9* and *ARO10*. It has been demonstrated that overexpression of *ARO80* can upregulate the transcription levels of *ARO9* and *ARO10*, resulting in a significant increase in the 2-PE titre (Kim et al., 2014). Similarly, modification of the GATA factor can also effectively improve the efficiency of 2-PE synthesis; for example, overexpression of *GLN3* and *GAT1* in *S. cerevisiae* YS58 led to an increase in 2-PE production (Wang et al., 2018).

Additionally, recent studies have shown that mepanipyrim and tetraconazole residues could affect the biosynthesis of volatile aromatic compounds during the winemaking process (Sieiro-Sampedro et al., 2019a,b). In particular, tetraconazole seems to accelerate the Ehrlich pathway, and several genes of the Ehrlich pathway (*BAT1*, *PDC1*, *PDC5*, *ADH1*, and *SFA1*) are upregulated in tetraconazole-enriched medium. Therefore, the activity of aminotransferase, decarboxylase, and dehydrogenase may be enhanced by tetraconazole (Sieiro-Sampedro et al., 2020).

### The Effect of L-Phe Catabolism in the Ehrlich Pathway

In the Ehrlich pathway, L-Phe is transaminated to phenylpyruvate, and α-ketoglutarate is the acceptor of the amino group and converted into glutamate, followed by the conversion of phenylpyruvate into 2-PE. Glutamate, a crucial intermediate, is converted into glutamine by glutamate synthase or glutamate-ammonia ligase, and then, purine and pyrimidine are synthesized to maintain cell growth (Figure 3). In addition, glutamate is used for the biosynthesis of other amino acids, such as alanine, methionine, leucine, phenylalanine, serine, and proline. Glutamate and glutamine are the hubs of nitrogen metabolism; 85% of cellular nitrogen is derived from the amino nitrogen of glutamate, and the remaining 15% is derived from the amide nitrogen of glutamine (Ljungdahl and Daignan-Fornier, 2012). As a preferred nitrogen source, glutamate may inhibit the transport and catabolism of L-Phe when it accumulates in yeast cells. However, glutamate is the byproduct of L-Phe catabolism *via* the Ehrlich pathway, which may lead to the accumulation of glutamate.

To avoid the accumulation of glutamate and alleviate the inhibition of L-Phe utilization, glutamate is deaminated to produce α-ketoglutarate and NH_4_^+^ by NAD-dependent glutamate dehydrogenase or by NADP-dependent glutamate dehydrogenase encoded by *GDH2* and *GDH3*, respectively. This conjecture is consistent with the results of a previous study: the expression levels of *GDH2* and *GDH3* are upregulated in *S. cerevisiae* CEN.PK113-7D with L-Phe as the sole nitrogen source (Boer et al., 2007). In addition, the reaction resupplies α-ketoglutarate and NADH for L-Phe catabolism *via* the Ehrlich pathway. Therefore, when *S. cerevisiae* is cultured with L-Phe as the sole nitrogen source, intracellular glutamate undergoes rapid synthesis and catabolism to support cell growth and biosynthesis.

α-Ketoglutarate is another important substrate of the Ehrlich pathway, and its intracellular concentration also affects the efficiency of 2-PE synthesis from L-Phe. In addition, α-ketoglutarate is a crucial intermediate of the tricarboxylic acid cycle (TCA). Therefore, α-ketoglutarate is the converging point of L-Phe catabolism and glucose metabolism. α-Ketoglutarate is modulated by the retrograde regulation (RTG) pathway, which mainly regulates the expression of *CTT*1/2, *IDH*1/2, and *ACO*1 (Giannattasio et al., 2005). In the presence of a poor nitrogen source, the genes related to the synthesis of α-ketoglutarate are upregulated by the RTG pathway to meet the demand of α-ketoglutarate (Broach, 2012). Therefore, with L-Phe as the sole nitrogen source, the expression of *CTT*1/2, *IDH*1/2, and *ACO*1 might also be upregulated to satisfy the needs of the Ehrlich pathway.

## Conclusion and Perspectives

*Saccharomyces cerevisiae* is generally recognized as safe (GRAS) and is typically used in food and industrial production. *Saccharomyces cerevisiae* can also serve as one of most promising microorganisms for the biosynthesis of natural 2-PE. *Saccharomyces cerevisiae* recognizes extracellular L-Phe through the SPS-sensing pathway, regulates the expression of genes that are critical for the transport of L-Phe, and then recognizes intracellular L-Phe through the TOR-sensing pathway. Finally, L-Phe is converted into 2-PE *via* the Ehrlich pathway. The currently known transporters involved in the transport of L-Phe are Agp1p, Bap2p, Bap3p, and Gap1p.

However, the four permeases contributing to L-Phe transmembrane transport could be finely tuned based on different nitrogen sources. When *S. cerevisiae* Y294 is grown with ammonium salt as the sole nitrogen source, the harvested cells are transferred to the buffer system containing L-Phe and leucine, in which Bap2p is the principal L-Phe transporter. Agp1p is the major transporter of L-Phe when *S. cerevisiae* Y294 *gap1*∆ is cultured in synthetic medium. The transporters of L-Phe are regulated synergistically by different pathways. For example, Bap2p is regulated simultaneously by the SPS-sensing pathway and GAAC pathway, and Agp1p is regulated simultaneously by the SPS-sensing pathway and NCR. The details of the regulatory mechanism of L-Phe transport remain unclear, and further study is necessary to determine how the regulatory pathways synergistically regulate the transport of L-Phe.

Although, we have gained a certain understanding of the catabolism and regulation of L-Phe, the highest production of 2-PE by the Ehrlich pathway reached 6.3 g/L through reconstruction of the metabolic module. However, 2-PE induces high levels of toxicity in yeast cells, which is the biggest bottleneck to the process for improving 2-PE production (Jin et al., 2018; Dai et al., 2020). *In situ* product removal, two-phase extraction and *in situ* product adsorption are used to alleviate the toxicity of 2-PE, which has been shown to be effective but uneconomical. Many efforts have been made to screen robust strains (Lu et al., 2016; Dai et al., 2020; Zhan et al., 2020). However, the molecular mechanism of 2-PE tolerance remains unclear. Recently, Hap5 was discovered to be a necessary regulator of 2-PE resistance in *Candida glycerinogenes* (Wang et al., 2020). Therefore, further study on the toxicity of 2-PE in yeast cells would lead to the mechanism of 2-PE tolerance, which would provide more theoretical guidance for further increasing the production of 2-PE.

L-Phenylalanine has been proven to be a nonpreferred nitrogen source in *S. cerevisiae*, and the crucial genes of the Ehrlich pathway are modulated by NCR in the presence of preferred nitrogen sources, blocking synthesis of 2-PE on the industrial scale. The details of the regulatory mechanism should be further studied to avoid repression to augment the efficient synthesis of 2-PE.

Although good results were achieved with L-Phe as the precursor (Wang et al., 2019), the high price of L-Phe might be a good economic barrier for the scaled-up application of this bioprocess. *Saccharomyces cerevisiae* possesses the native ability to synthesize 2-PE from glucose *via* the phenylpyruvate pathway, which is very complex, contains many branches competing for carbon flow and is strongly feedback-inhibited by L-Phe. Therefore, the efficiency of the phenylpyruvate pathway is very low (Hassing et al., 2019; Wang et al., 2019). To reduce the cost, the development of cheaper substrates or robust strains might be the best alternative to accomplish industrial production. Many efforts have been made to improve the *de novo* synthesis of nonconventional yeast or develop robust strains. It has been demonstrated that engineered *Pichia pastoris* can produce 2-PE from simple sugars, and the concentration of 2-PE increased to 1,169 mg/L through genetic engineering strategies (Kong et al., 2020). Moreover, 2-PE can be synthesized by solid-state fermentation using low-cost raw materials by *Pichia kudriavzevii*, and the maximum 2-PE titre was 27.2 mg per gram of dry substrate (Martinez-Avila et al., 2020). Interestingly, hydrolysed corn stover or molasses can be used as a carbon source to produce 2-PE by nonconventional yeast or *Bacillus licheniformis*, with higher 2-PE concentrations of 3.67 g/L and 4.41 g/L, respectively (Mierzejewska et al., 2019; Zhan et al., 2020). To reduce the cost, in current studies, *Escherichia coli* has been developed for the biotransformation of L-Phe, glucose, or glycerol to 2-PE with resting cells, leading to a higher 2-PE concentration (9.1 g/L) from glucose or glycerol (Liu et al., 2018; Lukito et al., 2019; Sekar et al., 2019).

Understanding the meticulous process of L-Phe metabolism and current research will facilitate the ultimate industrial production of 2-PE, 2-PEA, and other valuable derivatives of 2-PE. Moreover, branched-chain amino acids (leucine, valine, and isoleucine), aromatic amino acids (tyrosine, and tryptophan), and sulfur-containing amino acids (methionine) are assimilated by the Ehrlich pathway to produce other higher alcohols, such as 2-methylbutanol, propanol, and 3-methylbutanol. This research could play a guiding role in the production of other higher alcohols.

## Author Contributions

XC and CY conceived the review. JD and HX wrote the manuscript. All authors contributed to the article and approved the submitted version.

### Conflict of Interest

The authors declare that this study received funding from China Tobacco Corporation and Hubei tobacco company. The funder was not involved in the study design, collection, analysis, interpretation of data, the writing of this article or the decision to submit it for publication.
